# Pain Control Disparities in Acute Pancreatitis

**DOI:** 10.7759/cureus.27507

**Published:** 2022-07-31

**Authors:** Michelle Knees, Ellen Sarcone, Audrey Goold, Jonathan Mroch, Julie Knoeckel

**Affiliations:** 1 Division of Hospital Medicine, University of Colorado Anschutz Medical Campus, Aurora, USA; 2 Division of Hospital Medicine, Denver Health Hospital Authority, Denver, USA; 3 Department of Medicine, Denver Health Hospital Authority, Denver, USA; 4 Information Technology, Denver Health Hospital Authority, Denver, USA

**Keywords:** substance use disorder, acute pancreatitis, pain scores, pain disparities, pain control

## Abstract

Background and objective

Patient treatments and outcomes have historically differed based on age, sex, race/ethnicity, and social factors, and there is a growing awareness that such disparities still exist. While prior studies have found that patients belonging to minority groups have their pain undertreated, few studies have evaluated pain control based on age, sex, body mass index (BMI), or presence of a substance use disorder (SUD). The studies that do exist have inconsistent results. This study aimed to evaluate pain control in patients admitted to a Denver academic safety net hospital for acute pancreatitis. Pancreatitis is an inherently painful condition involving pancreatic inflammation and for which adequate pain control is a cornerstone of treatment; this makes it an ideal disease state for an exploratory analysis into the experience of pain within different patient groups.

Methods

This was a retrospective cohort study of patients treated at the Denver Health Medical Center from January 1, 2017, through December 31, 2019, for acute pancreatitis; 659 patients met the inclusion criteria and were included in the study. Pain control during the first 24 hours of hospital admission was analyzed by comparing controlled vs. uncontrolled reports of pain and mean pain scores. Patients were stratified by age, sex, self-reported race/ethnicity, BMI, and presence of SUD at the time of admission. Achievement of "controlled pain," as defined by a pain score below the patient’s stated functional pain goal, was then analyzed. Chi-squared analysis was employed to look into differences within and between groups. Additionally, a t-test was used to compare mean pain scores between groups with controlled and uncontrolled pain.

Results

A statistically significant difference in pain control was found when stratified by age or the presence of SUD (p<0.001). Within these groups, 39% of those aged 18-40 years achieved pain control, compared with 49% of those aged 41-64 years and 66% of those aged 65 years and older. Among those with active SUD, only 41% were able to achieve pain control compared with 58% of those without SUD. Among those who achieved pain control, the average mean pain score was 5, which decreased to 4 within 24 hours. Among those who did not achieve pain control, the average mean pain score was 7, which remained at 7 at 24 hours (p<0.001).

Conclusions

We did not find significant differences in the ability to achieve tolerable pain control based on sex or BMI. We were unable to appropriately analyze differences based on race/ethnicity due to an inability to differentiate between White Hispanic and White non-Hispanic populations within Epic. However, we did find significantly poorer pain control in younger patients and those with an active SUD.

## Introduction

Treatment and outcomes for a variety of medical conditions have historically differed based on race and ethnicity [1-9]. There is increasing awareness that these disparities persist, especially in the treatment of pain. A large literature review found that, in comparison to non-Hispanic Whites, racial minorities consistently receive less pain control in both acute, chronic, and palliative care settings [1]. Similarly, a cross-sectional analysis of children treated in emergency rooms for acute appendicitis found that Black patients were less likely to receive any analgesia for moderate pain and less likely to receive opioids for severe pain [2].

However, there is sparse literature on whether there is a correlation between age, sex, BMI, or presence of a substance use disorder (SUD) and patient-reported adequate treatment of pain, and the literature that does exist provides contradictory findings. For example, one study found that men are perceived as less willing to report pain and that women are perceived as more sensitive and less able to endure pain [3]. Conversely, another study found that, in a sample vignette, male patients were more likely to be rated as having higher pain and their pain was treated more quickly [4]. Another large study evaluated patients referred for chronic pain management from 2014 to 2017 and found that obesity, female gender, and belonging to the age group of 45-56 years were more likely to be correlated with inadequate pain management in chronic pain conditions [5]. Research on pain control in patients with SUD is still in an early stage. While there are studies that have looked at stigma and various treatment modalities for those with SUD, there is minimal literature evaluating whether patients with a current history of SUD report adequate pain control during inherently painful conditions, such as acute pancreatitis.

This study sought to evaluate local pain control disparities in patients admitted to the Denver Health Medical Center for acute pancreatitis. Acute pancreatitis, an inherently painful condition involving pancreatic inflammation, is uniquely suited for a study on patient-reported pain control as it is a clinical condition for which analgesia is a cornerstone of management [10,11]. We explored if there are differences in patient-reported pain control in acute pancreatitis depending on several patient characteristics that may impart risk for inequitable care.

This article was previously presented as a poster at the Society of Hospital Medicine Converge 2022 annual meeting on April 8, 2022.

## Materials and methods

This study was reviewed by the Colorado Multiple Institutional Review Board (COMIRB) and deemed exempt from requiring IRB approval. We designed a retrospective cohort study and, using the Epic Clarity database, reviewed patients treated at the Denver Health Medical Center from January 1, 2017, through December 31, 2019, for acute pancreatitis. Of the 1,565 patients admitted for pancreatitis during this period, 659 met the inclusion criteria and were subsequently analyzed. The inclusion criteria were as follows: patients aged ≥18 years, an International Classification of Diseases, Tenth Revision (ICD-10) admission diagnosis of acute pancreatitis or acute on chronic pancreatitis, initial admission to a general medical floor, at least one stated functional pain goal within 24 hours, and two or more verbal pain scores recorded in the pain assessment nursing flowsheet within the first 24 hours of admission. The exclusion criteria were as follows: patients aged <18 years, less than two recorded pain scores during the initial 24 hours of admission, <1 stated functional pain goal within 24 hours of admission, admission or transfer to the medical ICU during the first 24 hours of admission, ICD-10 admission diagnosis of only chronic pancreatitis, active pregnancy or incarceration at the time of admission, or deceased at the time of chart review.

Patients were stratified into the following groups and subgroups: sex assigned at birth (male, female); age (18-40, 41-64, ≥65 years); self-reported race/ethnicity (White non-Hispanic, Black, Hispanic, Asian, American Indian/Alaska Native, other/unknown); BMI (<18.5, 18.5-29.9, 30-39.9, ≥40); and presence of active SUD at the time of admission (present, absent) as defined by ICD-10 F10-F19 substance use billing codes. The local nursing workflow includes asking patients for a functional pain score goal, which is a pain score that correlates with a tolerable pain level for that patient. To analyze the primary outcome of interest (controlled vs. uncontrolled pain), a dichotomous variable was coded to indicate whether a patient reached their numerically reported goal of a tolerable pain score (functional pain goal) within the first 24 hours of hospital admission. "Tolerable pain" was defined as ≥1 verbal pain score equal to or less than the patient’s highest stated functional pain goal. Chi-squared tests were used to assess levels of statistical significance both within and between groups. Additionally, a t-test was used to compare mean pain scores between groups with controlled and uncontrolled pain, and a chi-squared test was used to compare the administration of opioids.

## Results

We identified statistically significant differences in pain control at 24 hours based on age and for patients with an active SUD (p<0.001). We also observed statistically significant differences in the lowest mean self-reported pain score at 24 hours between patients who achieved pain control and those who did not (Table 1).

**Table 1:** Comparison of patient groups and subgroups who were able to achieve target pain score (controlled pain) and those who did not achieve target pain score (uncontrolled pain) Statistical significance (p<0.001) was seen in terms of patient "age" and "SUD" groups as well as between mean pain scores SD: standard deviation

Encounter details	P-value	Total sample	Controlled pain	Uncontrolled pain
N		659	315	344
Age, years, mean ± SD		48 ± 14	51 ± 15	46 ± 13
Active SUD disorder	p<0.001	N=659	N=315	N=344
Yes		402	166 (41%)	236 (59%)
No		257	149 (58%)	108 (42%)
Age categories	p<0.001	N=659	N=315	N=344
18-40 years		206	80 (39%)	126 (61%)
41-64 years		379	186 (49%)	193 (51%)
65+ years		74	49 (66%)	25 (34%)
Sex	p=0.68	N=659	N=315	N=344
Male		390	189 (48%)	201 (52%)
Female		269	126 (47%)	143 (53%)
Race/ethnicity	p=0.36	N=659	N=268	N=308
White/Hispanic		475	225 (47%)	250 (53%)
Black		101	43 (43%)	58 (57%)
Asian		7	4 (57%)	3 (43%)
American Indian/Alaska Native		11	5 (45%)	6 (55%)
Other/unknown		65	38 (58%)	27 (42%)
BMI	p=0.82	N=610	N=284	N=326
<18.5		42	17 (40%)	25 (60%)
18.5-29.9		435	204 (47%)	231 (53%)
30-39.9		112	52 (46%)	60 (54%)
40.0+		21	11 (52%)	10 (48%)
Average pain scores	p<0.001			
Pain score #1, mean ± SD		6 ± 3.1	5 ± 3.5	7 ± 2.0
Pain score #2, mean ± SD		6 ± 3.3	4 ± 3.5	7 ± 2.4

**Figure 1:**
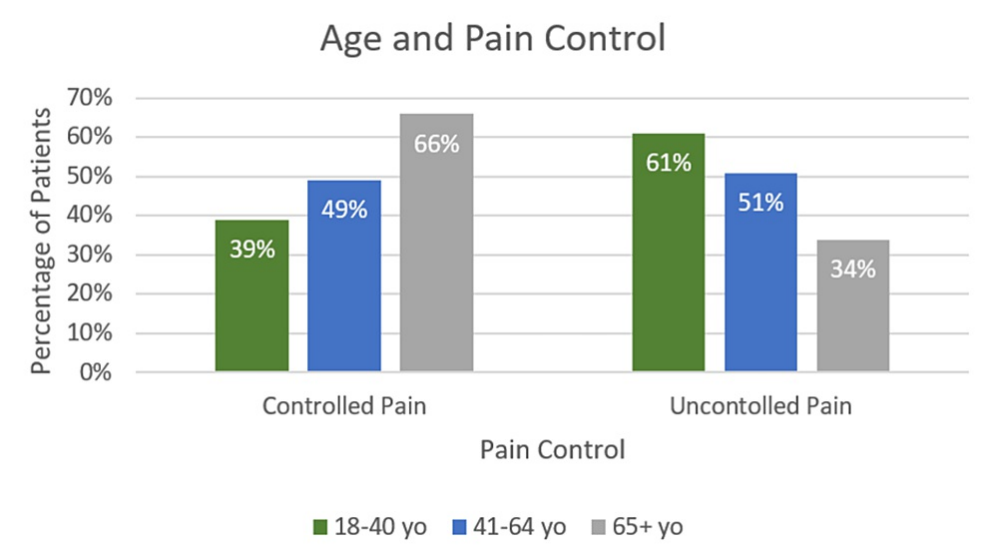
Association between age and pain control A statistically significant difference (p<0.001) was found in the ability to achieve the target pain score based on patient age

Of those aged 18-40 years, 61% reported uncontrolled pain at 24 hours, compared with 51% of those aged 41-64 years and 34% of those aged 65 years and older (Figure 1).

**Figure 2:**
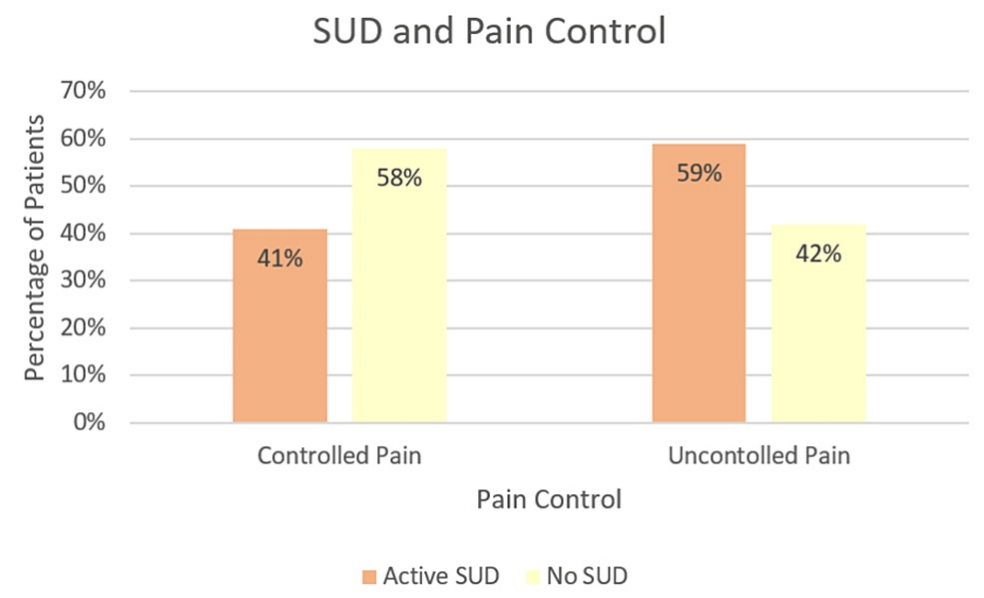
Association between SUD and pain control A statistically significant difference (p<0.001) was found in the ability to achieve the target pain score based on the presence of SUD SUD: substance use disorder

Among those with SUD, 59% reported uncontrolled pain compared with 42% of those without SUD (Figure 2). We included all patients with an ICD-10 code for SUD within our SUD cohort (n=402); 86.8% of these patients had an alcohol use disorder, 11.2% had multiple concurrent SUDs (i.e., alcohol and opioids), 1% had marijuana use disorder, 0.7% had opioid use disorder, and 0.2% had a non-alcohol, opioid, or marijuana use disorder. Patients with SUD comprised 61% of the study population and, among those with SUD, 88% received opioids within the first 24 hours of hospitalization.

Among those who did not achieve pain control, the initial pain score was 7 ± 2.0; it remained at 7 ± 2.4 at 24 hours. Among those who achieved pain control, the average initial pain score was 5 ± 3.5, which decreased to 4 ± 3.5 after the intervention. This was statistically significant (p<0.001).

## Discussion

We did not find significant differences in the ability to achieve tolerable pain based on race or ethnicity, despite numerous studies reporting that belonging to a minority race correlates with poorer pain control. However, these findings are severely limited by our inability to differentiate between White Hispanic and White non-Hispanic patients within Epic. Our EHR requires multiple variables to capture the Hispanic population, including stratifying patients into "Hispanic," "Latino," and "Mexican" with significant portions of this population also being coded as "White." We, therefore, combined White non-Hispanic and White Hispanic patients into one ethnic group during analysis, which may have obscured the disparities within this group as well as between the other racial and ethnic groups. Additionally, we were underpowered to detect differences in our Asian and American Indian/Alaskan populations. We also did not find differences in the ability to achieve target pain scores based on patients' sex or BMI.

We did find a correlation between active SUD and worse pain control. The majority (86.8%) of our patients with SUD had alcohol use disorder. Other studies have found that excessive alcohol use is associated with greater pain severity, likely through a combination of psychosocial effects and anatomical and biochemical changes, which may partly explain the difficulty with adequate pain control [12]. Within the SUD group, 88% received opioids within the first 24 hours of admission, suggesting that difficulty with pain control may not just be physician reluctance to prescribe opioids to those with SUD. We did not quantify daily morphine milligram equivalents between populations, and hence we could not assess whether opioid medications were dosed and titrated differently between groups.

We also found that younger age correlates with worse pain control. Prior studies have found conflicting results regarding the effect of age on pain, with some studies finding that older age corresponds to an increased pain threshold and other studies noting decreased pain thresholds [9,13,14]. Within our sample, patients with SUD were, on average, three years younger when compared to the overall sample of all patient encounters; SUD may be a confounding factor when looking at age and pain control. 

Of note, pain across all patient demographics remained difficult to control with 40-60% of almost all stratified patient groups reporting uncontrolled pain. The only groups with <40% poorly controlled pain were patients over the age of 65 years and patients not receiving opioids; better pain control in the group not receiving opioids likely speaks to the fact that pain was relatively well-controlled to begin with, thus negating the need to escalate to an opioid pain control strategy.

Our study has several limitations. As mentioned above, we were unable to differentiate between White Hispanics from White non-Hispanics. Similarly, we did not assess for differences in pain control among patients who do not speak English as their primary language. An additional limitation is small numbers within some cohort groups, which may have underpowered our ability to detect differences between groups. We also assessed pain control by relying solely on numerical patient-reported pain scores. Pain scores are subjective, difficult to standardize across patients, and can be affected by factors outside of acute pain intensity (i.e., chronic pain, cognitive patterns, coexisting mental health disorders). Interpretation of adequate pain control based only on verbal pain score is likely inadequate [15]. Similarly, we were unable to assess whether physicians utilized these pain scores during treatment decisions, such as titration of pain medication or decision to discharge. Finally, our study was a single-center analysis and was only designed to identify differences in the ability to achieve target pain control at our institution. It was not designed to investigate possible etiologies of differences in the ability to control pain, including physician bias, cultural perceptions of pain, central sensitization or opioid tolerance, and confounding factors such as chronic pain.

Additional avenues of study should include qualitative analyses of physician and patient experiences in treating pain, rapidity of opioid titration, amount of morphine equivalents administered, and use of pain adjuncts and addiction medicine consults with the goal of achieving both better pain control and less variability between patient groups.

## Conclusions

Within our local institution, we did not find differences in the ability to achieve self-reported “goal pain” scores depending on patient sex or BMI; however, we did find a difference in the ability to achieve goal pain based on age and presence of an active SUD. We were unable to adequately assess for differences based on race and ethnicity due to our inability to differentiate between White Hispanic and White non-Hispanic patients. This study highlights the need for healthcare institutions to analyze their local populations to identify disparities in pain control and ultimately find ways to address these differences.
